# Radiotherapy Plus Concomitant and Adjuvant Temozolomide for Glioblastoma—A Critical Review

**DOI:** 10.4137/cmo.s390

**Published:** 2008-05-21

**Authors:** Ricardo J. Komotar, Marc L. Otten, Gaetan Moise, E. Sander Connolly

**Affiliations:** Department of Neurosurgery, Columbia University, New York, NY

Glioblastoma Multiforme (GBM), the most common primary brain tumor in adults, has a median survival rate of 12 months, even with aggressive resection and radiation therapy (Fig. 1A). Regrettably, outcomes for this disease have not substantially improved since the Brain Tumor Study Group published their results over 25 years ago. The current standard of care for GBM consists of surgery followed by adjuvant radiotherapy. Although numerous phase III trials have attempted to demonstrate the benefit of employing chemotherapy in these patients, either concomitantly with radiotherapy or as isolated adjuvant treatment, a significant prolongation of survival has never been documented.

Stupp and colleagues investigated the use of temozolomide (Fig. 1B) as an adjuvant to radiotherapy in patients with GBM and recently published their findings in The New England Journal of Medicine (1). In this multi-institutional randomized clinical trial, 573 patients with newly diagnosed, histologically confirmed GBM received either radiotherapy alone or radiotherapy plus concomitant temozolomide. Results demonstrated a median overall survival of 14.6 months (95% CI 13.2–16.8) for patients treated with radiotherapy and chemotherapy, compared to 12.1 months (95% CI 11.2–13.0) for those treated with radiotherapy alone. More importantly, the two-year survival rate was 26.5 percent (95% CI 21.2–31.7) for patients treated with radiotherapy and chemotherapy, compared to only 10.4 percent (95% CI 6.8–14.1) for those treated with radiotherapy alone. The calculated hazard ratio for death in the radiotherapy-plus-temozolomide group was 0.63 (p < 0.001). Of note, only 7 percent of patients receiving concomitant chemotherapy experienced grade III/IV hematologic toxic effects. Based on these findings, the authors concluded that the addition of temozolomide to radiotherapy for GBM leads to survival benefits that are both clinically and statistically significant with minimal additional toxicity. It is important to note, however, that patients with poor preoperative neurological status undergoing biopsy only are unlikely to benefit. Moreover, it remains unclear whether the addition of chemotherapy increases the risk of radiation induced cognitive deficits.

In the same issue of The New England Journal of Medicine, Hegi and colleagues investigated the relationship between promoter methylation of the MGMT DNA-repair gene and responsiveness to temozolomide (2). The authors demonstrated that the survival benefit of temozolomide is limited to those patients with tumor containing methylated MGMT promoters. Among these patients, the median survival with radiotherapy and temozolomide was 21.7 months (95% CI 17.4–30.4), compared to 15.3 months with radiotherapy alone (95% CI 13.0–20.9; p = 0.007). An insignificant survival difference was demonstrated between treatment groups in patients with unmethylated MGMT promoters. This finding points to a genetic basis for the efficacy of temozolomide and underscores the role of gene expression in optimal patient selection.

Although these articles represent a substantial step forward in the treatment of GBM, at this point it is too early in standard clinical practice to choose treatment according to molecular criteria alone. It is also important to remember that several factors likely contributed the studies’ findings. First, the majority of patients was otherwise healthy and had favorable prognostic variables—age less than 70 years, good performance status, and tumor debulkment. Second, temozolomide is an alkylating agent. As compared to the previously tested nitrosourea drugs, this type of agent may be more effective and less toxic when administered concurrently with radiotherapy. Looking ahead, the cost of this treatment may bring a considerable financial burden to the health care providers of developing economies. To this end, until available tests can readily identify the methylation status of the MGMT promoters, not all patients should be referred for this therapy. Nevertheless, these studies are important contributions to the literature, supporting radiotherapy combined with temozolomide as the new platform from which innovative regimens for treating malignant gliomas are explored.

## Figures and Tables

**Figure 1:**
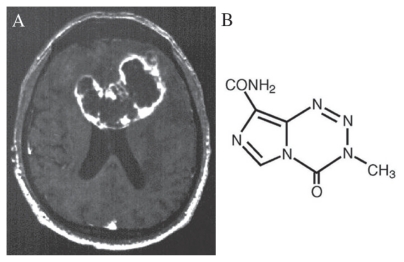


## References

[1] Stupp (2005). Radiotherapy plus concomitant and adjuvant temozolomide for glioblastoma. N. Engl. J. Med..

[2] Hegi (2005). MGMT gene silencing and benefit from temozolomide in glioblastoma. N. Engl. J. Med..

